# Gandan Oral Liquid Improves Exudative Pneumonia by Upregulating Bacteria Clearance via Regulating AQP5 and MUC5AC in Rats

**DOI:** 10.1155/2022/3890347

**Published:** 2022-04-26

**Authors:** Yinlong Wang, Jing Yan, Peijia Wang, Xiaoqin Xu

**Affiliations:** College of Veterinary Medicine, Yangzhou University, Yangzhou 225009, China

## Abstract

Gandan oral liquid (GOL) is a mixture of crude extracts from licorice and Radix isatidis. Clinically, it has been widely used in the treatment of exudative pneumonia (EP) in animals. But the molecular mechanism of these effects is unclear. Therefore, antibacterial activity and therapeutic effect were tested in vitro and in vivo. Exudative pneumonia was established with the intraperitoneal injection of LPS, followed by continuous intranasal inoculation of *Klebsiella pneumoniae* (*KP*). After that, Gandan oral liquid, acetylcysteine, and levofloxacin were given through the intragastric route for five days, and clinical symptoms were observed and counted. The bacterial content of alveolar lavage fluid was determined, hematology analysis was performed, and lung histology examination was performed. Western blotting, immunohistochemistry, and immunofluorescence were used to detect the expression levels of AQP3, AQP5, and MUC5AC in lung tissues. ELISA kit was used to detect serum and BALF cytokines levels. The results showed that GOL (242 mg/mL) had no antibacterial activity on *Klebsiella pneumonia* (*KP*), and the effect was significantly worse than levofloxacin. However, the therapeutic test in vivo of the rat model of bacterial EP showed different results. After treatment, GOL administration ameliorated EP and increased the expression of mucoprotein -5AC (MUC5AC), and GOL promoted water secretion of the respiratory tract by increasing the expression of aquaporin-5 (AQP5) and decreasing the levels of proinflammatory cytokines (TNF-*α*, IL-6, and IL-1*β*). *Conclusion*. GOL accelerates the water secretion of respiratory tract, inhibits the inflammatory response, induces removal of bacteria of respiratory tract via the AQPs/MUC pathway, and ultimately ameliorates EP.

## 1. Introduction

Exudative pneumonia is a nonclassical medical term used to describe inflammation of the lungs characterized by a large inflammatory exudation of the alveoli and bronchi [1–3]. EP is the basis for the pathological development of many lung diseases, which play a key role in the disease course of chronic obstructive pulmonary disease (COPD) [4] and acute respiratory distress syndrome [5]. EP can be caused by a variety of factors, mainly bacterial or virus infections [6], but long-term smoking or exposure to smoke and dust can also increase the risk of lung tissue infection [6–8], which can lead to severe pneumonia that can lead to shock and even death.

At present, the treatment of EP mainly adopts antibiotic therapy [9]. However, according to “the ACCP evidence-based clinical practice guidelines 2006” and “Guidelines for Diagnosis, Treatment of Cough 2015,” only 10.95% of respiratory tract inflammation belongs to bacterial infection. The use of large amounts of antibiotics is ineffective and promotes the development of bacterial resistance.

In recent years, with the vigorous development of complementary and alternative medicine, the enthusiasm for exploring new natural plants for the treatment of EP has increased exponentially. GOL is a mixture of crude extracts from licorice and Radix isatidis, and the main active substances are glycyrrhizin, licorice glycoside, glycosylamine, lignan, 2,4 (1H,3H)-quinazoline dione, apigenin, and indirubin [10, 11], which exhibited abundant pharmacological effects, including antibacterial, antiviral, anti-inflammatory, antioxidant, antitumor, and immunomodulatory [11–16]. However, the underlying molecular mechanism in regulating mucus dilution and anti-inflammatory has not been fully revealed.

Microbial clearance is the basic therapy for bacterial or virus pneumonia. Aquaporins-5 (AQP5) is expressed in rat respiratory epithelial cells, which dominate the water metabolism of cells and are closely related to the formation of respiratory exudates [17]. AQPs decreased with the increase of MUC expression in the inflammatory process of the pneumonia rat model, and AQPs increased with the decrease of MUC expression after the improvement of animal respiratory symptoms after treatment, so Gao speculated that AQPs/MUC might mediate the formation of respiratory mucus [18]. High concentrations of mucus in the airways play a key role in the pathologic process of mucociliary damage and airway obstruction, leading to airflow obstruction and providing a focus for bacterial infection [19]. While explaining the resistance of the animal body to respiratory tract infection, JA Whitson believed that in addition to inhibiting microbial activity, the respiratory tract of the body also forms mucus to carry microorganisms out of the body, so as to achieve the purpose of microbial removal [20].

In this study, based on an assessment of the protective effect of GOL on lung injury, the effect of GOL on AQPs/MUC was further investigated, which might provide a deeper comprehension of GOL for overexpression of respiratory mucus.

## 2. Materials and Methods

### 2.1. Materials

In GOL, the main ingredients are licorice and Radix isatidis. Each 100 mL is equivalent to 24.4 g of raw medicine, produced by Santel (Inner Mongolia) Technology Co., Ltd. The following were used: levofloxacin hydrochloride, 0.1 g/tablet according to C_18_H_20_FN_3_O_4_ (Hunan Dino Pharmaceutical Co., Ltd.); acetylcysteine, 0.2 g/tablet (Hainan Zambon Co., Ltd.).

### 2.2. Animals

Sixty SPF Wistar male rats (body weight (200 ± 10 g)) were purchased from the Comparative Medicine Center of Yangzhou University. The approval number of the animal experiments in this study was DWLL-202012004. All animal experiments were performed in accordance with the guidelines approved by the Animal Care and Use Committee of Yangzhou University, according to the experimental animal 3R principle. The experimental animal permit license was issued by the Science and Technology Department of Jiangsu Province in China (SYXK[Su]2017-0044). Animal experiments were supervised and inspected by the Animal Welfare and Ethics Committee. Animal carcasses, tissues, or body fluids were centralized for pollution-free treatment.

### 2.3. In Vitro Antibacterial Investigation

*Klebsiella pneumonia (KP)*, ATCC 1705, and *Escherichia coli*, ATCC 25922, were used. Single colonies were isolated from McConkey culture medium and placed in LB broth enrichment solution. The colonies were amplified on a shaker at 37°C, 120 RPM. OD value was measured each 2 h, and the bacteria were counted, and the growth curve of the bacteria was recorded. The concentration of the original solution was as follows: GOL, 242 mg/mL; acetylcysteine, 100 mg/mL; levofloxacin, 50 mg/mL. Dilute the drug by gradient dilution to 1, 1/2, 1/4, 1/8, 1/16, 1/32, 1/64, 1/128, 1/256, 1/512, 1/1024, 1/2048, 1/4096, 1/8192, 1/16384, and 100 *µ*l per well. The logarithmic growth phase bacterial solution was added to each well and incubated in a 37°C incubator for 24 h. The bacteria were inoculated in MH medium and incubated in a 37°C incubator for 24 h to observe the growth of the bacteria. Minimum inhibitory concentration (MIC) is defined as the minimum concentration of a drug that reduces the number of bacteria growing by 50% compared with the blank control group.

### 2.4. Antipneumonia Effect on the Rat Model

Many researchers have successively reported the preparation of rat pneumonia model by using pneumonia model, including unilateral nasal dripping, surgical tracheal dripping, and tracheal dispersion [21–25], which achieved good results and made an important contribution to the study of pneumonia disease. However, the use of KP alone twice a week nasal drip needs more than eight weeks to form a model, the surgical tracheal dripping model only takes short time but strictly requires for the environment, equipment, and surgical skills. Meanwhile, it cannot well simulate the onset of natural pneumonia process. We decided to adopt a simple, rapid, and effective method to establish a rat model of exudative pneumonia. After intraperitoneal injection of 2 mg/kg LPS and bilateral nasal drip of 50 *µ*l 1 × 10^7^ CFU/mL *KP* for five days every day, the rat model of exudative pneumonia was established. After the success of the model was confirmed, the next experiment was carried out.

**Figure 1:**
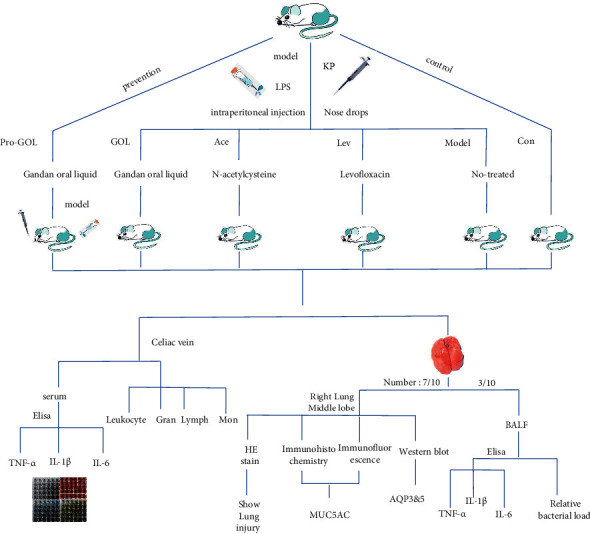
Experiment design scheme and the biological operation method used in the antipneumonia study.

Forty model rats with exudative pneumonia were randomly divided into four groups, including 290 mg/kg/d GOL treatment group, 15 mg/kg/d acetylcysteine treatment group, 6 mg/kg/d levofloxacin treatment group, and positive control group. Ten healthy rats were selected (90 mg/kg/d GOL prevention group). Another 10 rats were selected (blank control group). After five days of treatment, pentobarbital sodium was injected for deep anesthesia, the abdominal cavity was opened, and blood was collected and divided into EDTA anticoagulant tubes and nonanticoagulant tubes. The anticoagulant tubes were used for the classified count of white blood cells. Three rats in each group were randomly selected, and the bronchoalveolar lavage fluid was collected aseptically to obtain 1 ml BALF. Sterile centrifuge tubes was centrifuged at 3500 RPM at 4°C for 10 minutes, and the supernatant was obtained and placed at −80°C for ELISA. The other was used to measure the relative bacterial load of lung tissue. The remaining seven rats in each group were sacrificed for collecting the right middle lobe of the lung for histopathology, immunohistochemistry, immunofluorescence, and western blot detection (Figure 1).

### 2.5. Observation of Clinical Symptoms

We observed and analyzed the clinical data. For body temperature, we measured the rectal temperature with an electronic thermometer. The number of breaths was measured using abdominal fluctuation, and the time was 1 min. For the respiratory status, the rats in each group were observed for 30 min, and the severe respiratory disorder was measured once.

### 2.6. IL-1*β*, IL-6, and TNF-*α* Measurement

Nonanticoagulant tubes were centrifugated at 3000 rpm for 10 min for serum separation, which was then stored at −80°C for further detection. Three rats in each group were randomly selected, and the BALF was collected aseptically and centrifugated for supernatant collection. Then, the TNF-*α*, IL-1*β*, and IL-6 expression levels detection were performed with commercial ELISA kits (Mlbio Co., Ltd., China), following the instructions. The sample optical density (OD) value was determined using a UV spectrophotometer at 450 nm, and the concentration of inflammatory factors was calculated using the related standard curve.

### 2.7. BALF for Bacterial Counting

5 ml sterile normal saline (37°C) was infused into the distal trachea, 1 ml of BALF was collected, and the collected samples were immediately placed on ice and transferred to a −80°C ultralow temperature refrigerator within 1 h. For testing, BALF was diluted two times with sterile saline, and 200 *µ*l BALF diluent was inoculated in MACC medium for 36 h at 37°C. The bacterial growth was observed and counted.

### 2.8. Hematoxylin-Eosin, Immunohistochemistry, and Immunofluorescence Staining

Lung tissues were sampled and fixed in 4% paraformaldehyde and then embedded in paraffin. Then, the lung tissue blocks were sliced into 4 *μ*m sections.

For detection of inflammatory cell infiltration and the extent of lung injury, the sections were stained with hematoxylin and eosin (H&E) staining.

For immunohistochemistry, the tissue slides were dewaxed and rehydrated and then immersed in 0.01 M sodium citrate buffer (pH 6.0) for antigen retrieval with microwave and treated with 3% hydrogen peroxide in methanol to exhaust the endogenous peroxidase activity. Thereafter, tissue slides were blocked with PBS in 2% bovine serum albumin for 1 hour at room temperature (RT), and then, the MUC5AC antibody (diluted 1 : 400) was added for incubation overnight. Then, the sections were cleaned and incubated with the corresponding HRP-conjugated secondary antibodies at RT for 2 hours, and sections were developed using 3,3 ′- diaminobenzidine (DAB) for 10 min and counterstained with hematoxylin and fixed with resin.

For immunofluorescence staining, antigen recovery sections were blocked with 2% BSA for 1 h at RT and then incubated with MUC5AC antibody (diluted 1 : 200) overnight at 4°C. After washing three times with PBS, fluorescence-conjugated secondary antibodies (diluted 1 : 1,000) and 4′,6-diamidino-2-phenylindole (DAPI) were adopted for MUC5AC antibodies and nuclei staining, respectively.

Then, H&E and immunohistochemistry slices were photographed under bright field microscope (Nikon Corporation, Tokyo, Japan). Meanwhile, immunofluorescence staining sections were photographed with a fluorescence microscope photograph system (Lecia Corporation, Frankfurt, Germany). The sample images were analyzed and compared using ImageJ.

### 2.9. Western Blotting

Total proteins from lung tissues were extracted with precooled RIPA lysis buffer (BioChain Institute Inc., Hayward, CA), and BCA protein assay kit (Pierce Biotechnology Inc., IL) was used for quantification. Proteins were boiled with 4 × loading buffer for 5 min after the concentrations were adjusted. For analysis in western blots, protein samples were separated by 4–12% gradient SDS-PAGE gels and then transferred onto polyvinylidene difluoride (PVDF) membranes (Millipore, Biotechnology Inc). After blocking with 5% skim milk for 1.5 h at RT, the membrane was incubated in diluted primary antibodies overnight at 4°C. The following primary antibodies were purchased from Cell Signaling Technology, Danvers, MA: Aquaporin-5 (AQP5, #25682), Aquaporin-3 (AQP3, #26612), and Recombinant Mucin 5 Subtype AC (MUC5AC, #26718). Following this incubation, the membranes were incubated with species-specific horseradish peroxidase (HRP) conjugated secondary antibodies for 2 h. HRP signals were detected with chemiluminescence substrate. Moreover, *β*-actin was selected as the reference protein.

### 2.10. Statistical Analysis

All data were processed using SPSS 20.0 application software, and ANOVA was used for statistical processing. The results were expressed as mean ± standard deviation. *P* < 0.05 was considered significantly different, and *P* < 0.01 was considered extremely significantly different.

## 3. Results

### 3.1. GOL Has No Antibacterial Activity In Vitro

In vitro antibacterial experiments, we found that GOL (242 mg/mL) had no antibacterial activity against *KP*. The minimum inhibitory concentrations (MIC) of acetylcysteine and levofloxacin were 12.5 mg/mL and 1.2 × 10^−2^ mg/mL, respectively. In the clinical treatment experiment, the concentration of the drug was GOL (prevention), 45 mg/mL; GOL (cure), 145 mg/mL; acetylcysteine, 7.5 mg/mL; levofloxacin, 3 mg/mL. GOL and acetylcysteine did not show antibacterial activity in vivo. After 6 mg oral administration of levofloxacin in rats, 1 h later, the peak concentration (C max) of 8.2 × 10^−2^ mg/mL in blood was found, which was greater than the MIC in vitro. We believed that levofloxacin still had strong antibacterial activity in vivo.

### 3.2. GOL Reduces the Amount of Bacteria in the Lungs

**Figure 2:**
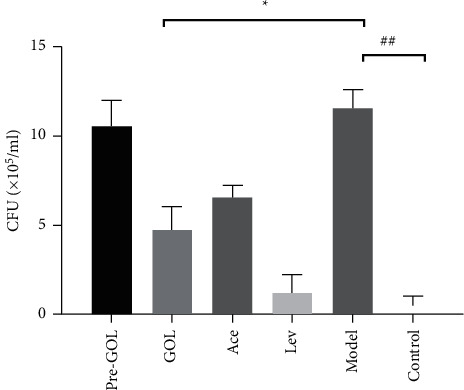
Bacterial load in bronchoalveolar lavage fluid. Pro-GOL: prevent exudative pneumonia rats treated with GOL. GOL: rats with exudative pneumonia treated with GOL. Ace: rats with exudative pneumonia treated with acetylcysteine. Lev: the exudative pneumonia rats treated with levofloxacin. Model: rats with exudative pneumonia which was not treated. Con: control rats with no pneumonia. The data are presented as means ± SD (*n* = 10). ^*∗*^*P* < 0.05; ^*∗∗*^*P* < 0.01.

Microbial clearance is the basic therapy for bacterial or virus pneumonia. We tested the bacterial load of *KP* in BALF, and the antibacterial activity of levofloxacin was much higher than that of GOL and acetylcysteine, almost eliminating bacteria. However, compared with the model group, GOL and acetylcysteine also showed a higher bacterial clearance rate (Figure 2).

### 3.3. GOL Reduces the Clinical Symptoms for the Therapy of Bacterial Pneumonia

**Figure 3:**
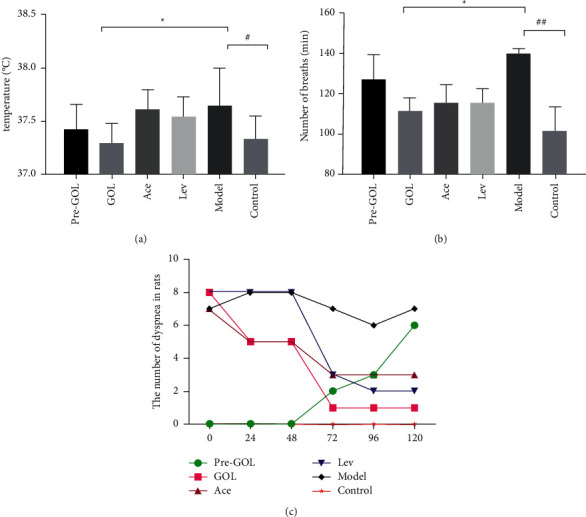
Effects of different treatment methods on clinical symptoms of rats (*n* = 10). (a) Anal temperature, (b) number of breaths, and (c) the number of dyspnea in rats. Pro-GOL: prevent exudative pneumonia rats treated with GOL. GOL: rats with exudative pneumonia treated with GOL. Ace: rats with exudative pneumonia treated with acetylcysteine. Lev: the exudative pneumonia rats treated with levofloxacin. Model: rats with exudative pneumonia which was not treated. Con: control rats with no pneumonia. Data are presented as means ± SD (*n* = 10). ^*∗*^*P* < 0.05; ^*∗∗*^*P* < 0.01.

After treatment, the body temperature of group GOL was significantly lower than that of group Model (*P* < 0.05), and there was no significant difference between group GOL and group Con (Figure 1(a)); The number of BPM (breaths per minute) in groups Ace and Lev was significantly lower than that in group Model (*P* < 0.01). There was no significant difference in BPM between group Pro-GOL and group Model, and it was significantly higher than that in group Con (*P* < 0.05) (Figure 3(b)). The number of rats with obvious cough and sneezing symptoms decreased from 8/10 to 1/10 in group GOL, which was lower than 6/10 in group Pro-GOL, 3/10 in group Ace, 2/10 in group Lev, and 8/10 in group Model (Figure 3(c)).

### 3.4. GOL Reduces the Immune Responses for the Therapy of Bacterial Pneumonia

After treatment, the total number of white blood cells in group GOL was significantly lower than that in group Model, and the lymphocyte percentage in group GOL was significantly lower than that in group Model, and there was no significant difference between group GOL and group Con, which was close to the trend in group Lev (Table 1).

Table 1 shows the effects of different treatment methods on blood cells. Pro-GOL: prevent exudative pneumonia rats treated with GOL; GOL: rats with exudative pneumonia treated with GOL; Ace: rats with exudative pneumonia treated with acetylcysteine; Lev: the exudative pneumonia rats treated with levofloxacin; model: rats with exudative pneumonia which was not treated. The data are presented as means ± SD (*n* = 10). ^*∗*^*P* < 0.05; ^*∗∗*^*P* < 0.01. Compared with group F, *P* < 0.05 #*P* < 0.05; ##*P* < 0.01.

**Figure 4:**
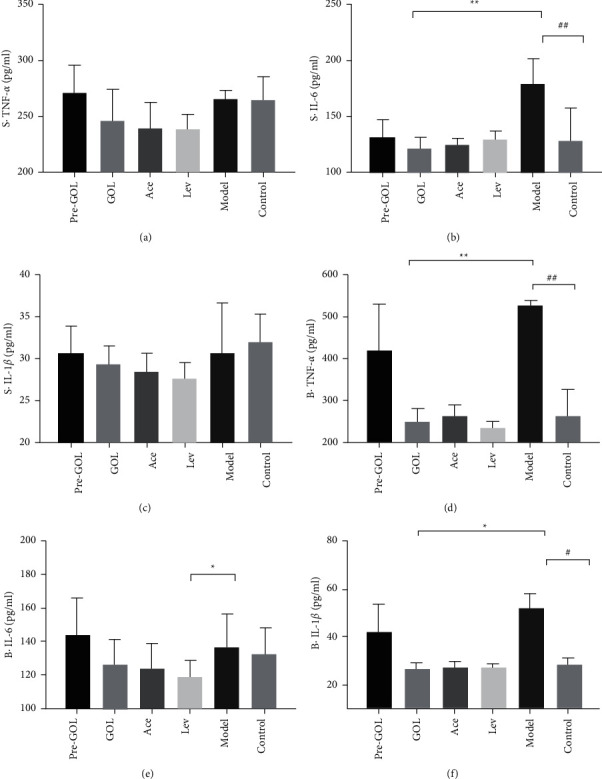
Effects of different treatments on the expression of TNF-*α*, IL-6, and IL-1*β*. The symbol “S ·” represents serum. The symbol “B ·” represent BALF. Pro-GOL: prevent exudative pneumonia rats treated with GOL. GOL: rats with exudative pneumonia treated with GOL. Ace: rats with exudative pneumonia treated with acetylcysteine. Lev: the exudative pneumonia rats treated with levofloxacin. Model: rats with exudative pneumonia which was not treated. The data are presented as means ± SD (*n* = 10). ^*∗*^*P* < 0.05; ^*∗∗*^*P* < 0.01.

The expression of IL-6 in the serum of group GOL was significantly lower than that of group Model (Figure 4(b)). The expression level of TNF-*α* in BALF was significantly lower than that in group Model (Figure 4(d)), and the expression level of IL-1*β* was significantly lower than that in group Model (Figure 4(e)), which was similar to that in group Ace and Lev (Figure 4(f)).

### 3.5. GOL Reduces the Bacteria-Induced Lung Injury

**Figure 5:**
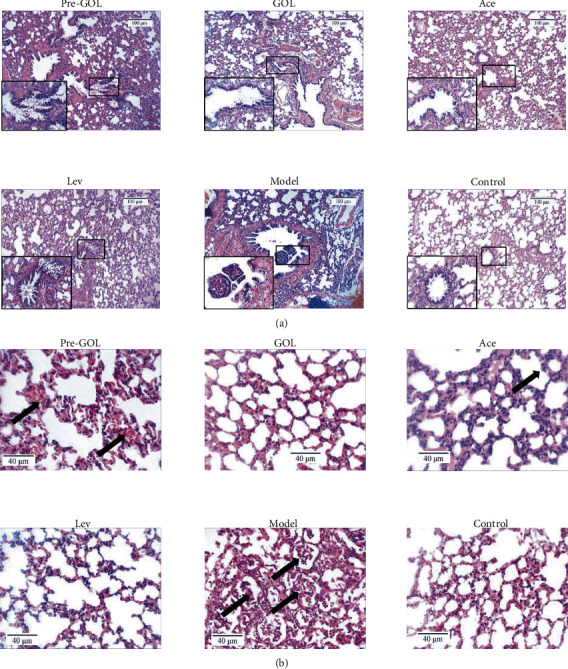
Lung histopathological section images (400x). Pro-GOL: prevent exudative pneumonia rats treated with GOL. GOL: rats with exudative pneumonia treated with GOL. Ace: rats with exudative pneumonia treated with acetylcysteine. Lev: the exudative pneumonia rats treated with levofloxacin. Model: rats with exudative pneumonia which was not treated. Taking the bronchus as the main field of vision (a). Taking the alveoli as the main field of vision (b). The arrow indicates the location of the obvious lesion.

In the study, GOL remarkably alleviated the *KP* pneumonia-induced rat lung injury. The result was confirmed using histopathological sections. In the lung tissue of rats, rupture of bronchial mucosa was observed, parts of columnar epithelial cells were exfoliated, alveolar structure was disarranged in different sizes, alveolar walls were thinning, and a large number of inflammatory exudates were observed in alveolar cavities (Figure 5(a)). The lung with nonpneumonia was control rats (Figure 5(b)). The exudation of lung tissue was reduced and the structure of bronchial mucosa was improved by GOL (Figure 5(b)).

### 3.6. GOL Downregulates the MUC5AC Expression in Bacterial Pneumonia

**Figure 6:**
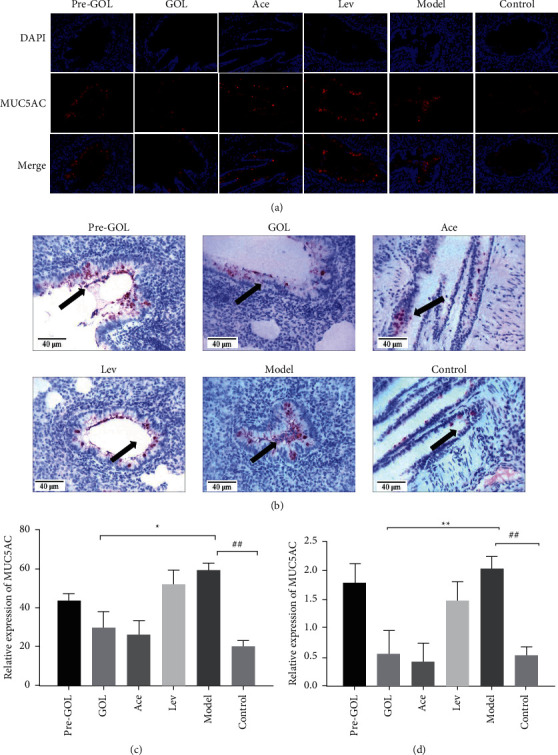
Immunofluorescence images (400x). Pro-GOL: prevent exudative pneumonia rats treated with GOL. GOL: rats with exudative pneumonia treated with GOL. Ace: rats with exudative pneumonia treated with acetylcysteine. Lev: the exudative pneumonia rats treated with levofloxacin. Model: rats with exudative pneumonia which was not treated. The arrows indicate MUC5AC expression. Immunofluorescence (a, c). Immunohistochemical (b, d). The data are presented as means ± SD (*n* = 10). ^*∗*^*P* < 0.05; ^*∗∗*^*P* < 0.01

In the fluorescence results, we found a large number of MUC5AC in the alveolar and bronchial epithelial cells of the bacterial infected rat lung tissue (Figure 6(a) and 6(c)). In contrast, GOL and acetylcysteine significantly reduced the overexpression of MUC5AC (*P* < 0.05) (Figures 6(b) and 6(c)). The MUC5AC protein expression levels were not significantly downregulated in Pro-GOL and Lev group (Figures 6(a) and 6(d)). It is known that persistent or repeated stimulation of inflammatory cytokines often leads to persistent overexpression of MUC5AC, further damaging inflammatory tissues. Therefore, GOL can effectively reduce the expression of MUC5AC before bacterial pneumonia causes lung injury. The immunochemical results showed similar trends as immunofluorescence (Figure 6), but the decrease in MUC5AC was more pronounced after GOL and acetylcysteine (Figures 6(b) and 6(c)). This difference may have been caused by different test methods.

### 3.7. GOL Upregulates the AQP5 Expression in Bacterial Pneumonia

**Figure 7:**
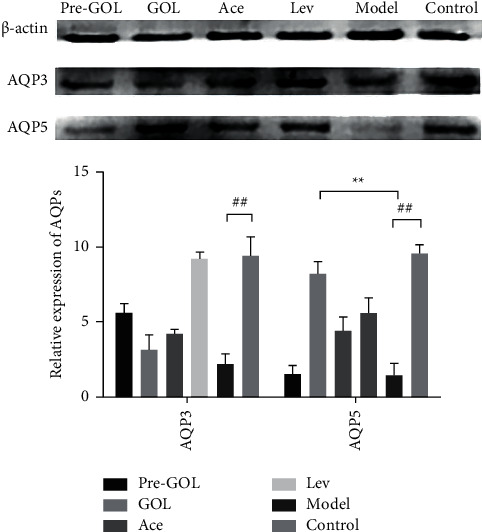
The western blot results of each group. Pro-GOL: prevent exudative pneumonia rats treated with GOL. GOL: rats with exudative pneumonia treated with GOL. Ace: rats with exudative pneumonia treated with acetylcysteine. Lev: the exudative pneumonia rats treated with levofloxacin. Model: rats with exudative pneumonia which was not treated. The data are presented as means ± SD (*n* = 10). ^*∗*^*P* < 0.05; ^*∗∗*^*P* < 0.01.

Generally, AQP3 and AQP5 are highly expressed in the lung tissue of nonpneumonic rats, which is also shown in this study (Figure 7(Con)). In particular, the expression of AQP3 and AQP5 in the lung tissues of the bacterial infected rats was significantly reduced (Figure 7(Mod)). In contrast, levofloxacin significantly increased the expression of AQP3 and AQP5 (Figure 7(Lev)). GOL significantly increased the expression of AQP5 (Figure 7(GOL)). Acetylcysteine also increased the expression of AQP5 (Figure 7(Ace)). However, Pro-GOL increased the expression of AQP3, but not significantly. Therefore, GOL can effectively promote the expression of AQP5 before bacterial pneumonia causes lung injury.

## 4. Discussion

In China, natural plants such as Radix isatidis and licorice have been used for many centuries as effective drugs for the treatment of respiratory tract inflammation. In this study, the rat model of exudative pneumonia was established by intraperitoneal injection of LPS and continuous bilateral nasal drop *Klebsiella pneumoniae*. Our team studied the effect of GOL on EP in a rat model and selected levofloxacin and acetylcysteine as the treatment control group. After five days of GOL treatment, we found that the respiratory symptoms of the animals were significantly improved. GOL inhibit the inflammatory response in serum and BALF and increased the expression of AQP5 in lung tissues and decreased the expression of MUC5AC. GOL reduce the amount of bacteria in lung tissue and reduce the damage caused by *Klebsiella pneumoniae* to lung tissue. In summary, our study suggests that GOL has a therapeutic effect on EP.

Aquaporins (AQPs) were first discovered on the erythrocyte membrane of mammals by Agre in 1988; AQP3 and AQP5 are expressed in large quantities in lung tissues [26]. AQPs exist in the cell membrane with homologous tetramer structure. Water molecules do not combine with AQPs; they cross the cell membrane only by osmosis and proton repulsion [13], which greatly improves the efficiency of water metabolism. When mammalian epithelial cells were cultured alone, knockout of AQPs gene reduced cell water transport rate by 80%–90% [27, 28]. In animal experiments, knockout of AQPs gene in rats also reduced the overall water transport rate by 50%–60% [29], indicating that AQPs plays a crucial role in the process of water transport in animals. This has also attracted the attention of researchers studying EP. The typical symptom of EP is large quantities of inflammatory exudates in bronchi and alveoli. The relationship between the formation of these inflammatory exudates and AQPs and their interaction mechanism have become a hot topic of many scholars' research. Studies have shown that when AQPs gene is knocked out in mice, the permeability of alveolar capillary in mice is significantly decreased [30]. It has been proved that the deletion of AQP5 gene can lead to the aggravation of acute lung injury induced by *Pseudomonas aeruginosa* in mice [31]. During the establishment of acute lung injury model in rats, the expression level of AQPs in lung tissue of model group was significantly decreased, while the expression level of TNF-*α* and IL-6 in lung tissue was significantly increased, while the expression level of TNF-*α* in treatment group was significantly decreased and AQPs expression level was significantly increased [32]. These results suggest that aquaporin is involved in the inflammatory process of lung tissue and may be negatively correlated with the inflammatory process and the expression of some inflammatory factors.

Mucoprotein (MUC) is a kind of glycoprotein mainly secreted by epithelial goblet cells. Under normal circumstances, MUC5AB is the main component of respiratory mucus, but it is mainly secreted by MUC5AC in pathological conditions [33]. MUC5AC expression is positively correlated with the progression of respiratory inflammation, regulating the production and secretion of MUC5AC through NF-*κ*B and IL-13-STAT6-SAM signaling pathways [33, 34]. The potential role and regulatory mechanisms of MUC5AC in chronic obstructive pulmonary disease remain unclear. These results indicate that MUC5AC, like AQP3 and AQP5, is involved in the inflammatory process of lung tissues and may be positively correlated with the inflammatory process and the expression of some inflammatory factors.

TNF-*α* -AQPS-MUC5AC regulating shaft (TAMR) is an imaginary model to explain the regulation of mucus secretion of respiratory tract. At present, there is no clear evidence of upstream and downstream regulation relationship between TNF-*α*-AQPs-MUC5AC regulating shaft (TAMR). The expression of AQPs and MUC5AC was significantly decreased when TNF-*α* secretion was increased. In addition, MUC5AC will be correspondingly decreased when AQPs expression level increases after treatment [35]. MUC5AC synthesis and secretion increased by 57.9% and 85.3%, respectively, after five days of AQPs gene knockout in airway glands [18]. Scholars have predicted the existence of TAMR based on causality. However, the specific relationship remains to be further studied by scholars.

Previous studies showed that the extracts of licorice and Radix isatidis had good antibacterial activity. In this study, GOL (242 mg/mL) showed no antibacterial activity against *Klebsiella pneumoniae*, which may be related to the different drug concentrations used. However, in this study, GOL still showed a high pulmonary bacterial clearance rate (69.23% on average), which was lower than levofloxacin (92.30% on average) and close to acetylcysteine (53.84%), which may be the main reason for the therapeutic effect of GOL. Previous studies have shown that when acetylcysteine is used to treat respiratory tract inflammation with high exudation, it can promote the cleavage of mucin, dilute the exudate, and discharge the exudate out of the body, so as to achieve the purpose of bacterial clearance [36], which is also reflected in the results of this experiment. This study also found that GOL increases the expression level of AQP5 and decreases the expression level of MUC5AC in the lung tissues of pneumonia rats, which was similar to the results of Gao's study [18], indicating that GOL could act on the epithelial cells of lung tissues, improve the process of water metabolism, promote the elimination of respiratory mucus, and achieve the effect of alleviating the clinical symptoms of the disease.

In conclusion, we found that GOL improved respiratory tract symptoms, accelerated bacterial clearance, inhibited inflammatory response, and alleviated lung injury in rats with EP. It is possibly through the AQPs/MUC pathway and ultimately improved EP. Based on these studies and their clinical application, we believe that GOL can be used as a novel drug to treat EP. In the future, our team will further explore its mechanism of action and carry out clinical studies.

## Figures and Tables

**Table 1:** Determination of the blood routine test.

White cell	Pro-GOL	GOL	Ace	Lev	Model	Control
WBC (x10^9^)	7.03 ± 1.27^*∗*^	6.84 ± 0.91^*∗*^	6.40 ± 1.00^*∗*^	6.67 ± 0.81^*∗*^	9.35 ± 1.98	5.30 ± 1.02^*∗∗*^
Lymph (%)	71.09 ± 3.12	75.85 ± 3.35^*∗*^	69.02 ± 4.53	76.53 ± 4.49	72.82 ± 3.69	69.65 ± 3.78
Mon (%)	2.85 ± 0.55	2.46 ± 0.41	2.61 ± 0.23	2.39 ± 0.39	2.34 ± 0.59	2.02 ± 0.52
Gran (%)	26.06 ± 3.02	21.69 ± 3.03^*∗*^	28.37 ± 4.67	21.08 ± 4.48^*∗*^	24.84 ± 5.70	28.33 ± 3.64

## Data Availability

All original information of this study is included in the article. Further details are available from the corresponding author.
